# Usage of Cornea in Tympanoplasty: A Prospective Study 

**DOI:** 10.22038/ijorl.2025.84081.3831

**Published:** 2025

**Authors:** Sphoorthi Basavannaiah

**Affiliations:** *Department of ENT, Subbaiah Institute of Medical Sciences, Shimoga-577222, Karnataka, India.*

**Keywords:** Cornea, Graft, Middle Ear Surgery, Tympanic Membrane, Tympanoplasty, Temporalis Fascia

## Abstract

**Introduction:**

CSOM is inflammation of the mucoperiosteal lining of the ME cleft characterized by ear discharge, permanent perforation of the TM, and hearing impairment. If perforation fails to heal, surgical closure is done by M-plasty (Type-I T-plasty). The graft material used to reconstruct the eardrum is mainly TF. The goal is to reconstruct the TM and sound-conducting mechanism in a long-lasting way. Here, in this study, corneal homograft was considered for closure of TM perforation as part of primary T-plasty. To utilize unused cornea as homograft in T-plasty for closure of TM perforation and obtain acoustic qualities similar to normal TM.

**Materials and Methods:**

63 pts with TTD of ME in a hospital setup over a period of 2 years were considered for the study. All underwent T-plasty with use of Cornea and results were interpreted based on operative graft uptake and effective audible acoustics.

**Results:**

88% of patients showed successful corneal graft uptake, while 12% of patients with failed corneal graft uptake were planned for revision surgery with other graft materials. A literature review was done with a comparison in terms of various graft materials used to date, success of graft uptake, and audibility achieved following closure of TM perforation.

**Conclusion:**

Corneal graft has shown fairly significant results in terms of efficacious graft uptake and efficient hearing acoustics after T-plasty.

## Materials and Methods

Study design: Prospective follow-up study.

Place of study: The study will be conducted at *****.

Study period: 2 years.

Selection criteria: All 63 pts with TTD of ME attending the outpatient department of ***** from October 2018 to September 2020 were taken up for surgery after history and detailed clinical examination as shown in attachments 1 and 2. The study was conducted after obtaining Ethical clearance as shown in attachment 6. The Ethics Committee gave the official approval for the study in June 2020, which was delayed due to COVID, but the unofficial approval was obtained way before, after which the study was commenced. Also, the study involved merging both the ear and eye departments at different setups and multiple levels of agreement, which further delayed obtaining the approval. As this study involves association with the Eye Hospital, where the IRB is situated and the approval of the study was held and taken. In addition, the study also involved the permission from the Transplantation of Human Organs and Tissues Act with the Certificate number of Registration-JD/(H)/HOTA/19/2017-2018 for granting permission for approval on Eye Bank and Keratoplasty, plus the Certificate of Registration for Keratoplasty and Scleral Tissue Transplantation for Tympanoplasty with Registration-JD(M)/HOTA/32/2017-2018. While the informed consent for the study was taken from the patients as per attachment 3,4,5 and is provided as an attachment, which is usually the followed protocol at the hospital as part of the pre-operative checklist procedures for all emergency and elective cases.

Material used: Cornea/Sclera not utilised for optical keratoplasty that is rejected in the eye banks is used as the graft material as per attachment 7, 8. 

It is a simplified method to make use of a corneal disc preserved in glycerine for closure of TM perforation. Here, the perforated drum is reconstructed using a corneal graft by the inlay method through a transcanal incision under local anesthesia.

*Inclusion criteria:*

- Adult male & female between 18 and 60 years of age.
- Simple TM perforation with inactive ME mucosa.
- TM perforation with no acute or chronic ME disease.
- Ear to be dry for a minimum of 3 months.
- Otitis media with normal facial nerve function.
- Pts with conductive or mixed hearing loss on audiogram.

*Exclusion criteria:*

- Acute/chronic or active ME disease at the time of surgery.
- Any acute or chronic nose/throat infections preoperatively for at least 3 months.
- Children are excluded from the study.
- Any alterations of hematological parameters.

Procedure of the study**: **63 pts with TTD of ME attending the outpatient department of ***** from October 2018 to September 2020 were taken up for surgery after a history and detailed clinical examination, and a provisional diagnosis was arrived at. All necessary investigations were done to attain a final confirmatory diagnosis -

1. Otomicroscopic examination/oto-endoscopy—to confirm the provisional diagnosis after the initial clinical evaluation and find out additional information pertaining to the diagnosis: middle ear mucosa, status of the ossicular chain and its patency, status of the round and oval windows along with the round window reflex, and taking a swab of ear discharge and sending it for culture and sensitivity for choice of antibiotics pre- as well as post-procedure.
2. Pure Tone Audiometry (PTA)—to know the type and degree of hearing loss as per the table mentioned below: if conductive, sensorineural, or mixed hearing loss, to assess the air-bone gap. The audiogram is one of the important prerequisites as part of medicolegal importance. The audiometry was done following standard protocol and audiogram in every selected pt for the frequencies: 250, 500, 1000, 2000, 3000, 4000, 6000, and 8000 Hz after control of otorrhoea and in pts selected for surgical intervention. For calculating average air conduction threshold, 3 frequencies (Hz) were selected, which are 500, 1000, and 2000 Hz (which are the usual hearing frequencies), and hearing level (dB HL) between 30 and 60 dB. These frequencies were selected as they represent the speech frequency range, and the presence of any elevation of threshold in these frequencies is considered clinically significant, and these hearing levels were selected as they are the most commonly affected decibels in hearing with middle ear conditions, especially CSOM of TTD. PTA has become the standard behavioral procedure for describing audiometry sensitivity and used in this study for assessment of hearing level. In this study, pts with mostly the conductive type of hearing loss were essentially considered. The manual audiometer (Elcon N3D) with Carhart and Jerger’s technique (technique of 5 up and 10 down method) was implemented.
3. The test was performed in an acoustically treated room with no ambient noise. Standard headphones were used for air conduction. Whenever the interaural bone gap was 40 dB or more, masking was applied. Pt was explained about the procedure before audiometry and adequate time was taken for testing. Criteria for hearing improvement or hearing recovery after surgical intervention is mentioned in the Results section after the table of Audiometric results.

***Degrees of hearing loss (dB HL)***

- Normal (0-25): No perceived difficulty in hearing
- Mild (26-40): Difficulty in hearing &/or understanding quiet conversations, especially in noisy environments
- Moderate (41-60): Difficulty in hearing &/or understanding conversational speech
- Severe (61-80): Difficulty in hearing and/or understanding group conversations and loud speech
- Severe to Profound (81-90): Difficulty in hearing &/or understanding speech without hearing aids
- Profound (90+): Difficulty in hearing &/or understanding loud speech and sounds

1. X-Ray B/L Mastoid (Schuller’s view)—to know the type of mastoid air cell system: pneumatic, sclerotic, or diploic. If there is a plan to open the mastoid during the procedure of tympanoplasty in order to attain a functioning mastoid air cell system after disease clearance for acquiring good auditory properties. It is also one of the important prerequisites as per medicolegal importance.
2. HRCT Temporal bone- This radiological investigation is done as a mandate when there is a possibility of attico-antral disease to look for cholesteatoma: its extent and spread, structures engulfed/involved by the disease progression, and damage/destruction/erosion caused by the disease process: ossicles (malleus, incus, and stapes), round window, oval window, labyrinth, and hidden areas in the mastoid and temporal bone.
3. Eustachian tube function (ETF) tests - Diagnostic nasal endoscopy, tympanogram, and ear drop method (when asked in history) were performed to check for patent and functioning Eustachian tubes and to check for nasal conditions or any other pathology that is seen to compromise ETF. It is a very important prerequisite for successful graft uptake after T-plasty.

***Surgical Procedure:***

- Graft material: A corneo-scleral button obtained from Eye Bank is preserved in 15 ml of sterile glycerine and stored at room temperature (a corneo-scleral button can be preserved for up to 1 year).
- All pts were operated under local anesthesia with adequate sedation.
- Pre-medication was given to all pts taken up for surgery—2 ml of Inj. Phenargan (promethazine hydrochloride) and 1 ml of Inj. Fortwin (pentazocine).
- Following admission, a case sheet is filled by the surgeon, which includes surgical notes and the course of treatment during the stay at the hospital. The following procedure is undertaken once the authorization details are filled out by the pt.
- The following steps were followed while performing transcanal T-plasty using the inlay approach.
- Skin preparation is done by shaving hair 1 inch above and in front of the auricle of the ear to be operated.
- Painting with Betadine solution, following which the area was covered with sterile drapes. The ear canal was thoroughly cleaned with saline wash.
- Bleeding is reduced because of the absence of vasodilation caused by General Anaesthesia and the addition of vasoconstrictors.
- Facial nerve function and hearing can be checked on the table.
- Lidocaine hydrochloride (Xylocaine hydrochloride) along with 2% with 1:1,00,000 Epinephrine (adrenaline) solution is used for infiltration.
- Under a surgical microscope through an ear speculum larger than the opening of the ear canal, anesthetic solution is injected all along the bony-cartilaginous junction of the TM at the 12’O, 3’O, 6’O, and 9’O clock positions and the auriculotemporal nerve over the lateral and medial surfaces of the pinna.
- The edge of the perforation is removed using a sharp pick or sickle knife and cup forceps.
- Debridement of edges of the perforation (freshening of margins of perforation; separates continuity of inner mucosa with outer epithelium) ensuring that graft incorporates into TM remnant. Purpose—separates continuity of inner mucosa with outer epithelium and disrupts fistulous tract.
- The transmeatal incision is routinely used in stapes surgery.
- An incision is made through the largest ear speculum.
- A skin flap is created with radial incisions at 12, 6’O clock connected via an incision shaped like an inverted semilunar on the postero-superior bony wall of the canal just medial to the bony-cartilaginous junction.
- The tympanomeatal flap is raised atraumatically using a round angulated knife brought down to bone. The skin flap is then elevated with the Rosen elevator, pushing it towards the annulus. Once a plane of cleavage is developed, elevation is continued with a cotton ball moistened with adrenaline held by an alligator forceps.
- A grey bluish bulge indicates that the annulus fibrosus has been reached. A sickle knife or pick is pushed in between the annulus fibrous and bony rim, which lifts the annulus to create a tympanotomy. The opening into ME is widened by moving the instrument superiorly and inferiorly. The flap is then reflected forward. A cotton ball placed on the flap will prevent it from sliding back.
- The ME and ossicles are inspected and palpated to confirm ossicular continuity. (Middle ear disease is completely removed).
- Round window reflex and the Eustachian tube are inspected along with inspection of the undersurface of the TM.
- Canalplasty of anterior or posterior external auditory canal is performed to optimize visualization. The ME is packed with Gelfoam soaked in antibiotic ear drops and diluted adrenaline (1:10,000). Meso and hypotympanum are packed well.
- The area of tissue surrounding the perforation in contact with the graft is called the “bed” of the graft. The larger the bed and portion of graft in contact with it, the better are the chances of survival. The graft should be at least twice the size of the perforation.
- A slit is made in the superior aspect of the graft to accommodate placement of the graft medial to the handle of the malleus. Handle of the malleus is incorporated into the fibrous layer of the drum. The tightly adherent epithelial layer from the bone must be denuded.
- Epithelium migrates over newly formed connective tissue, leading to closure of perforation.
- The tympanomeatal flap is laid back down over the graft. Posterior canal skin edges are laid flat. Pieces of Gelfoam with antibiotic ointment are placed all along the TM, and the graft is layered laterally to cover vascular strip canal incisions. An antibiotic-coated ribbon gauge is placed in the external auditory meatus.

*Postoperative care:*

- Patient is discharged within 8 hours after a normal lunch and also once they can be ambulatory with normal vitals.
- Nil by mouth for 5 hours postoperatively.
- Conservative treatment is given: Broad-spectrum antibiotics, anti-inflammatories (Hifenac-D: aceclofenac, paracetamol & serratiopeptidase), and antihistamines (fexofenadine) for 7 to 10 days.
- First visit after 48 hours for metal pack removal. Advised topical antibiotic-steriod drops (Neomycin + Polymyxin + Hydrocortisone) once the pack is removed in order to dissolve the soaked gel foams.
- Follow-up visits after surgery weekly for 1 month. The ear canal is cleaned during F/U, and any remaining gelfoams are sucked out, and thereafter there are monthly visits for a minimum of 6 months for otomicroscopy.
- Pure tone audiometry is done based on the F/U of pts within 6 months, from 7 months to 1 year and after 1 year duration.
- The probable success rate of nearly 95% of graft uptake and postoperative pure-tone average air-bone improvement of nearly 16dB at usual hearing frequency is expected when compared to normal TM.

***Sample size calculation*** ***:***

$n=\frac{Z^{2}(\mathrm{pq})}{\mathrm{E2}}$

**n** = sample size, **Z** = standard error associated 

with chosen level of confidence (typically 1.96) (where Z² is the square of the confidence interval in standard error units), **p** = estimated percent in population (population proportion), according to previous studies or probable estimated success rates/estimated proportion of success, **q** = (1-p) or estimated proportion of failures, **E**² = margin of error/acceptable or allowable error (varies from 5-20%) [E2 is the square of the maximum allowance for error between true proportion and sample proportion]. In this study, 85-90% will be the expected success rate (i.e., 88% in this study) with a margin of error of 8%.

Z = 1.96, p= 88% (0.88), q= 12% (0.12), E = 8%

n = 1.96 X 1.96 X 0.88 X 0.12                    n=63                    0.0064 

Descriptive statistics will be calculated by using mean, standard deviation, median, and range (IQR), whichever is applicable. Frequency and percentages will be calculated. A comparison of hearing improvement before and after surgery will be done by using a paired t-test or Wilcoxon signed-rank test.

***Ethical considerations*** ***:***

- This study is a part of ongoing research to benefit the general population.
- Informed written consent is taken from parents/guardian of the patient.
- All examination findings are part of routine ENT practice and are not experimental.
- Confidentiality of records shall be maintained, and any disclosure of data, if the need arises, will purely be for legal reasons.
- There is no additional financial burden to the patient due to this study.
- There is no financial benefit from this study.

## Results

as shown in Table 1, 2.

**Fig 1:**
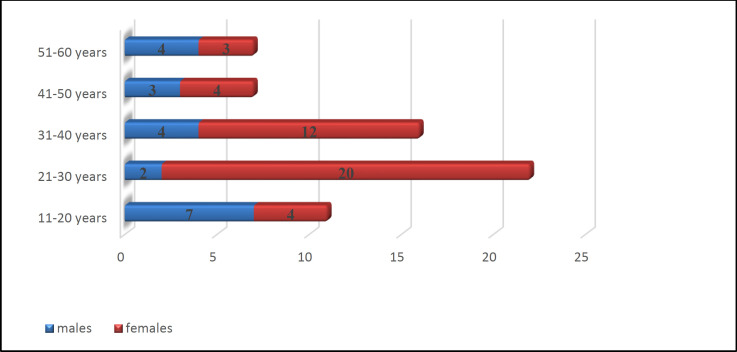
Gender distribution with age of the pts in the study.

**Fig 2:**
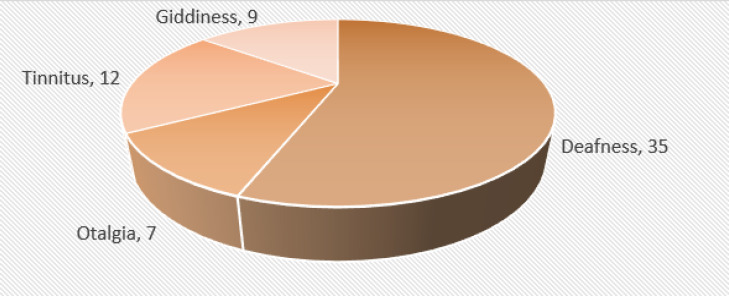
Primary symptomatology of presentation in all the pts in the study.

**Fig 3:**
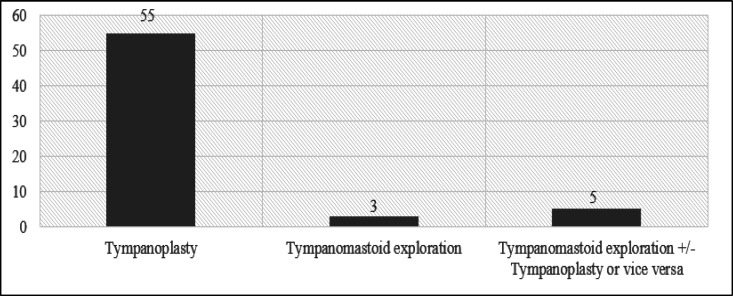
Various middle ear surgeries undergone by pts in the study.

**Fig 4:**
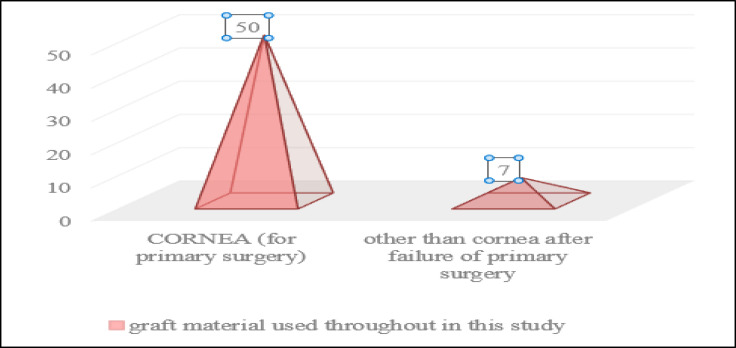
Graft material used throughout in the study to perform Tympanoplasty. The graft materials used for the revision cases after failure of corneal graft uptake following primary closure of tympanic membrane perforation. 2 pts each with temporalis fascia, tragal cartilage with skin & tragal perichondrium and 1 pt with tragal cartilage.

***Summary of this study as shown in Figure 1-4 & Tables 1 & 2:***

- 68% females and 32% males have been affected with this ME disease in this study of 63 pts. The ratio is nearly 2:1. 60% between 21 and 40 years of age showed TTD, while the rest, 40%, of the pts belong to the rest of the age groups, for a total of 63 pts.
- 52% of pts had a safe type of CSOM in the right ear, while 48% of pts in the left ear, in a total of 63 pts, with a barely substantial ratio among both groups.
- 87% of pts had U/L disease and 13% of pts had B/L disease among 63 pts.
- The ratio sums up to 7:1. 56% of patients had deafness as their primary complaint, while the rest, 44%, comprisedother symptoms of the ear, such as tinnitus, otalgia, and giddiness, as their primary complaint in 63 patients.
- 90% of patients are comprised of TTD of ME, while the other 10% belong to AAD.
- Overall, pts with ME disease in the study included 57 with TTD and 6 with AAD.
- The technique of grafting used in the study is the inlay method in 55 pts and the onlay method in 8 pts.
- 50 pts were with fresh cases for primary T-plasty, and 7 pts were revision cases who needed secondary closure in the study.
- Out of 63 pts, only 57 pts who underwent audiometric assessment postoperatively followed up earlier.
- 88% of pts showed successful corneal graft uptake, while 12% of pts with failed corneal graft uptake were planned for revision surgery with other graft materials.
- 83% is the total number of followed-up cases in this entire study (50 pts after primary surgery and 2 pts after secondary surgery).
- 11% had possible graft failure due to rejection, leaving behind residual perforation and otomycosis.
- The successful corneal graft uptake here in this study is 89% with a mean hearing gain of 16 dB at the usual hearing frequency when compared to various studies as quoted in the literature with very minimal complication rates.
- Criteria for successful corneal graft uptake considered in this study:

- Intact drum with generalized opacification, no perforation, and no retraction.
- Symptom resolution.
- Improved hearing.

- Total number of pts lost to F/U in the entire study is 11 pts, 6 after primary surgery and 5 following revision surgery after graft failure with corneal homograft. 52 pts F/U in the entire study. Hence, the success or failure of graft take-up/hearing improvement is unknown among non-F/U pts. Reasons for non-follow-up could be one or both of the mentioned possibilities: symptom resolution (yes/no) and hearing improvement (yes/no).
- As it is just a pilot study of one of its kind where corneal homograft was used as primary graft material for perforation closure. (At present, a similar study is ongoing with a large sample size, and very few pts have seen to not follow up as per the progression in the study).

**Table 1:** Audiometry assessment results in all pts who participated in the study

**+**	**Name of the** **Pt,** **sex &** **age**	**Symptom& ear operated**	**Pre-op audiogram findings (Hearing loss)**	**Post-op audiogram findings** **(Hearing in dB)**	**Preop AB gap** **(dB)**	**Postop AB** **Gap (dB)**	**A-B gap closure reduction/** **(hearing gain)**	**Duration after** **which the post-op** **audiogram done** **(mths/yrs)**
	MT 23/F	T (L)	Mod CHL	N	40	20	20	9 mths
	SK 19/M	Df (L)	Mild CHL	N	30	15	15	6 mths
	H 55/M	Df (R )	Mild CHL	N	15	0	15	7 mths
	R 50/F	Df (R )	Mild CHL	N	35	20	15	7 mths
	J 60/F	Df (R )	Mild CHL	N	15	10	5	5 mths
	R 18/F	Df (R )	Mod MHL	N	40	20	20	6 mths
	S 24/F	Df (L )	Mod CHL	N	35	15	20	7 mths
	D 39/M	G (L)	Mild CHL	N	20	15	5	6 mths
	PN 31/M	G(L)	Mild CHL	N	30	20	10	2 yrs
	G 35/F	G (L)	Mild CHL	N	25	5	20	8 mths
	A 18/M	G (R)	Mod CHL	n	40	20	20	5 mths
	P 32/M	O (R )	Mild CHL	N	25	10	15	4 mths
	A 28/F	T (L)	N	N	0	0	0	6 mths
	P 47/F	O (L)	Mod CHL	N	40	20	20	10 mths
	AN 18/M	G (R )	Mod MHL	Mild MHL	40	15	25	6 mths
	B 44/M	Df (L)	Mild CHL	N	30	10	20	5 mths
	L 55/M	Df (R )	Mild CHL	N	25	10	15	11 mths
	G 21/M	O (R )	Mod MHL	Mild MHL	40	15	25	7 mths
	UMS 26/F	Df (L)	Mild CHL	N	35	20	15	8 mths
	L 28/F	T (L)	Mild CHL	N	30	10	20	7 mths
	G 41/F	Df (L)	Mod CHL	N	40	20	20	5 mths
	A 20/M	Df (R )	Mod CHL	N	40	20	20	6 mths
	M 22/F	Df (R )	Mod CHL	N	40	20	20	6 mths
	S 18/F	Df (R )	Mod CHL	N	40	20	20	7 mths
	V 45/M	Df (R )	Mod CHL	N	40	20	20	9 mths
	R 23/F	Df (L)	Mild CHL	N	35	15	20	5 mths
	U 33/F	O (L)	Mild CHL	N	20	10	10	6 mths
	R 23/F	Df (R )	Mild CHL	N	10	0	10	2.5 yrs
	MJN 45/M	G (L)	Mild CHL	N	10	0	10	1.5 yrs
	GB 57/F	Df (L)	Mild CHL	N	25	5	20	6 mths
	S 26/F	G (R )	Mild CHL	N	10	0	10	1 yr 9 mths
	V 44/F	Df (R )	Mild CHL	N	15	10	5	4 mths
	R 25/F	Df (R )	Mild CHL	N	35	15	20	5 mths
	S 18/F	Df (R )	Mild CHL	N	25	5	20	1 yr 10 mths
	M 20/M	T ( L)	Mild CHL	N	15	0	15	6 mths
	RB 26/F	Df (L)	Mild CHL	N	25	10	15	1 yr 8 mths
	P 32/F	Df (R )	Mild CHL	N	15	5	10	6 mths
	S 60/F	T (R )	Mild CHL	N	15	5	10	6 mths
	B 35/F	Df (R )	Mod MHL	Mild MHL	40	10	30	6 mths
	SK 24/M	Df (R )	Mild CHL	N	20	10	10	6 mths
	S 60/M	T (R )	Mild CHL	N	15	5	10	5mths
	Y 38/M	Df (L)	Mild CHL	N	15	5	10	6 mths
	N 34/F	Df (R )	Mild CHL	N	15	10	5	6 mths
	M 31/F	Df (L)	Mild CHL	N	30	10	20	7 mths
	R 40/F	T (R )	Mod CHL	N	30	10	20	1 yr 9 mths
	F 38/F	G (L)	Mild CHL	N	30	10	20	7 mths
	V 19/M	Df (R )	Mild CHL	N	30	5	25	1 yr 4 mths
	T 29/F	T (R )	Mod CHL	N	45	25	20	6 mths
	S 19/F	G (L)	Mild CHL	N	30	10	20	1 yr 11 mths
	K 40/F	O (L)	Mod MHL	Mild MHL	40	10	30	8 mths
	G 40/F	T (R )	Mild CHL	N	35	15	20	11 mths
	SB 30/F	T (R )	Sev to prof MHL	Mild MHL	30	5	25	6 mths
	D 22/F	O (L)	Sev to prof MHL	Mild MHL	45	20	25	9 mths
	KSH 59/M	Df (L)	Mod CHL	N	30	15	15	1 yr 7 mths
	GK 21/F	Df (R)	Mild CHL	N	15	5	10	9 mths
	R 29/F	Df (L)	Mild CHL	N	15	5	10	1 yr 5 mths
	M 23/F	O (R )	Mod CHL	N	25	10	15	1 yr 3 mths

The statistical analyses are based on the audiometric assessment in 57 pts whose preoperative and postoperative audiometry are tabulated in Table 1 and attachments 9a and 9b.

63 pts were considered in the study who underwent ME surgery. 57 followed up with audiometric assessment. While 6 pts were lost to follow-up. Hence statistical analysis was derived for only 57 pts. 

Following surgery, audiometric assessment was done based on pt F/U within 6 months, from 6 months to 1 year and after 1 year. 12 pts F/U after 1 year. 18 pts F/U between 7 months and 1 year, and the rest, 27 pts, F/U within 6 months. 

Postoperative normal hearing was seen in 51 pts (89%), with the other 6 pts having mild MHL (due to the presence of SN factor involvement). 

The mean +/- SD of the preop AB gap is (27.7 +/- 10.8), the mean +/- SD of the postop AB gap is (11.3 +/- 6.65), and the mean +/- SD of the AB gap closure/hearing gain after surgery is (16.4 +/- 6.4). As per the results in this study, 89% pts showed complete recovery as per Siegel’s hearing recovery criteria following T-plasty.

*Siegel’s hearing recovery criteria:*

- Complete recovery: Final hearing better than 25 dB
- Partial recovery: Final hearing 25-45 dB; there is > 15 dB hearing gain.
- Slight recovery: Final hearing poorer than 45 dB, there is > 15 dB hearing gain.
- No recovery: Final hearing gain is poorer than 75 dB; there is less than 15 dB hearing gain.

***Statistical analysis:***

**Table:** Descriptive statistics

	N	Mean	Standard deviation	Minimum	Maximum	Percentiles		
						25^th^	50^th^ (Median)	75^th^
pre	57	27.37	11.145	0	45	15.00	30.00	40.00
post	57	9.65	6.399	0	20	5.00	10.00	15.00

**Table:** Wilcoxon signed ranks:

	N	Mean rank	Sum of ranks
post-pre-Negative ranks	56^a^	28.50	1596.00
Positive ranks	0^b^	00	00
	1^c^		
	57		

a: post< pre b: post > pre c: post = pre

**Table:** *Test statistics* ^b^

	post-pre
Z	-6.541^a^
Asymp. Sig (2 –tailed)	.000

a. Based on positive ranks b. Wilcoxon Signed ranks test

**Table 2:** Master chart of pts in the entire study

No	**Name of the patient**	**Age**	**Gender**	**Ear operated**	**Laterality**	**Symptoms**	**Technique used for graft placement**	**Disease of middle ear**	**Surgery underwent**	**Graft material used**	**F/U notes**	**Complications**
1	RS	29	F	R	B/L	dry ear, Df	Inlay	Tubotympanic disease	Tympanoplasty	cornea	graft ok	None
2	JN	45	M	L	U/L	giddiness	Inlay	Tubotympanic disease	1st: Tympanomastoid exploration, 2nd: Tplasty	1st: cornea, 2nd: temporalis fascia	graft ok	None
3	GB	57	F	L	U/L	dry ear, Df	Onlay	Tubotympanic disease	Tympanoplasty	cornea	graft ok	None
4	S	26	F	R	U/L	giddiness	Inlay	Tubotympanic disease	Tympanoplasty	cornea	graft ok	None
5	V	44	F	R	U/L	dry ear, Df	Inlay	Tubotympanic disease	Tympanoplasty	cornea	graft ok	None
6	RN	22	F	R	B/L	dry ear, Df	Inlay	Tubotympanic disease	Tympanoplasty	cornea	graft ok	None
7	S	18	F	R	U/L	dry ear, Df	Inlay	Tubotympanic disease	Tympanoplasty	cornea	graft ok	None
8	V	19	M	L	U/L	dry ear, Df	Inlay	Tubotympanic disease	Tympanoplasty	cornea	graft ok	None
9	M	20	M	R	U/L	dry ear, tinnitus	Inlay	Tubotympanic disease	1st: Tympanoplasty, 2nd: Revision Tympanoplasty	1st: cornea, 2nd: tragal perichondrium	graft failure	residual perforation
10	RB	26	F	L	U/L	dry ear, Df	Inlay	Tubotympanic disease	Tympanoplasty	cornea	graft ok	None
11	P	32	F	R	U/L	dry ear, Df	Inlay	Tubotympanic disease	Tympanoplasty	cornea	graft ok	None
12	S	60	F	R	U/L	dry ear, tinnitus	Inlay	Tubotympanic disease	Tympanoplasty	cornea	graft ok	None
13	B	35	F	R	U/L	dry ear, Df	Inlay	Tubotympanic disease	Tympanoplasty	cornea	graft ok	None
14	SK	24	M	R	U/L	dry ear, Df	Inlay	Tubotympanic disease	Tympanoplasty	cornea	graft ok	None
15	S	60	M	R	U/L	dry ear, tinnitus	Inlay	Tubotympanic disease	Tympanoplasty	cornea	graft ok	None
16	Y	38	M	L	U/L	dry ear, Df	Inlay	Tubotympanic disease	Tympanoplasty	cornea	graft ok	None
17	N	34	F	R	U/L	dry ear, Df	Inlay	Tubotympanic disease	Tympanoplasty	cornea	graft ok	None
18	M	31	F	L	U/L	dry ear, Df	Inlay	Tubotympanic disease	Tympanoplasty	cornea	graft ok	None
19	R	40	F	R	U/L	dry ear, tinnitus	Inlay	Tubotympanic disease	Tympanoplasty	cornea	graft ok	None
20	F	38	F	L	U/L	giddiness	Inlay	Tubotympanic disease	Tympanoplasty	cornea	graft ok	None
21	VN	20	M	R	B/L	dry ear, Df	Inlay	Tubotympanic disease	Tympanoplasty	cornea	graft ok	None
22	T	29	F	R	U/L	dry ear, tinnitus	Inlay	Tubotympanic disease	Tympanoplasty	cornea	graft failure	residual perforation
23	S	19	F	L	U/L	giddiness	Inlay	Tubotympanic disease	Tympanoplasty	cornea	graft ok	None
24	K	40	F	L	U/L	dry ear, otalgia	Inlay	Tubotympanic disease	Tympanoplasty	cornea	graft ok	None
25	G	40	F	R	U/L	dry ear, tinnitus	Inlay	Tubotympanic disease	Tympanoplasty	cornea	graft ok	None
26	SB	30	F	L	U/L	dry ear, tinnitus	Inlay	Tubotympanic disease	Tympanoplasty	cornea	graft ok	None
27	D	22	F	L	U/L	dry ear, otalgia	Inlay	Tubotympanic disease	Tympanoplasty	cornea	graft ok	None
28	KSH	59	M	L	U/L	dry ear, Df	Inlay	Tubotympanic disease	Tympanoplasty	cornea	graft ok	None
29	GK	21	F	R	U/L	dry ear, Df	Inlay	Tubotympanic disease	Tympanoplasty	cornea	graft ok	None
30	R	29	F	L	U/L	dry ear, Df	Inlay	Tubotympanic disease	Tympanoplasty	cornea	graft ok	None
31	M	23	F	R	U/L	dry ear, otalgia	Onlay	Tubotympanic disease	Tympanoplasty	cornea	graft ok	None
32	MT	23	F	L	B/L	dry ear, tinnitus	Inlay	Tubotympanic disease	Tympanoplasty	cornea	graft ok	None
33	S	19	M	L	U/L	dry ear, Df	Inlay	Tubotympanic disease	Tympanomastoid exploration	cornea	graft ok	None
34	H	55	M	R	U/L	dry ear, Df	Inlay	Tubotympanic disease	Tympanoplasty	cornea	graft ok	None
35	R	50	F	R	U/L	dry ear, Df	Inlay	Tubotympanic disease	Tympanoplasty	cornea	graft failure	residual perforation
36	J	60	F	R	U/L	dry ear, Df	Inlay	Tubotympanic disease	Tympanoplasty	cornea	graft ok	None
37	R	18	F	R	U/L	dry ear, Df	Onlay	Tubotympanic disease	Tympanoplasty	cornea	graft ok	None
38	S	24	F	L	U/L	dry ear, Df	Onlay	Tubotympanic disease	Tympanoplasty	cornea	graft ok	None
39	U	26	F	L	B/L	dry ear, Df	Inlay	Tubotympanic disease	Tympanoplasty	cornea	graft ok	None
40	D	39	M	L	U/L	giddiness	1st: Onlay, 2nd: Inlay	Tubotympanic disease	1st: Tympanoplasty, 2nd: Tympanomastoid exploration	1st: cornea, 2nd: temporalis fascia	graft failure	residual perforation
41	AN	35	F	L	U/L	dry ear, tinnitus	Inlay	Tubotympanic disease	Tympanoplasty	cornea	graft ok	None
42	P	31	M	R	U/L	giddiness	1st: Onlay, 2nd: Inlay	Tubotympanic disease	1st: Tympanomastoid exploration, 2nd: Tympanoplasty	1st: cornea, 2nd: tragal perichondrium	graft failure	residual perforation
43	G	33	F	L	U/L	dry ear, tinnitus	Inlay	Tubotympanic disease	Tympanoplasty	cornea	graft ok	None
44	G	35	F	L	U/L	giddiness	1st: Inlay, 2nd: Onlay	Tubotympanic disease	1st: Tympanoplasty, 2nd: Tympanoplasty	1st: cornea, 2nd: tragal cartilage	graft ok	None
45	A	18	M	R	U/L	giddiness	1st: Inlay, 2nd: Onlay	Tubotympanic disease	1st: Tympanoplasty, 2nd: Tympanomastoid exploration	1st: cornea, 2nd: tragal cartilage + skin	graft failure	residual perforation, otomycosis
46	P	32	M	R	U/L	dry ear, otalgia	Inlay	Tubotympanic disease	Tympanoplasty	cornea	graft ok	None
47	A	28	F	L	U/L	dry ear, tinnitus	Inlay	Tubotympanic disease	Tympanoplasty	cornea	graft ok	None
48	P	47	F	L	U/L	dry ear, otalgia	Inlay	Tubotympanic disease	Tympanoplasty	cornea	graft failure	residual perforation
49	A	18	M	R	U/L	giddiness	Inlay	Tubotympanic disease	Tympanomastoid exploration	cornea	graft ok	None
50	B	44	M	L	U/L	dry ear, Df	Inlay	Tubotympanic disease	Tympanoplasty	cornea	graft ok	None
51	L	55	M	R	U/L	dry ear, Df	Inlay	Tubotympanic disease	Tympanoplasty	cornea	graft ok	None
52	G	21	M	R	U/L	dry ear, otalgia	1st: Onlay, 2nd: Inlay	Tubotympanic disease	1st: Tympanomastoid exploration, 2nd: Tympanoplasty	1st: cornea, 2nd: tragal cartilage + skin	graft failure	residual perforation, otomycosis
53	U	30	F	R	B/L	dry ear, Df	Inlay	Tubotympanic disease	Tympanoplasty	cornea	graft ok	None
54	L	28	F	L	U/L	dry ear, tinnitus	Inlay	Tubotympanic disease	Tympanomastoid exploration	cornea	graft ok	None
55	G	41	F	L	U/L	dry ear, Df	Inlay	Tubotympanic disease	Tympanoplasty	cornea	graft ok	None
56	R	25	F	L	B/L	dry ear, Df	Inlay	Tubotympanic disease	Tympanoplasty	cornea	graft ok	None
57	A	20	M	R	U/L	dry ear, Df	Inlay	Tubotympanic disease	Tympanoplasty	cornea	graft ok	None
58	M	22	F	R	U/L	dry ear, Df	Onlay	Tubotympanic disease	Tympanoplasty	cornea	graft ok	None
59	S	18	F	R	U/L	dry ear, Df	Inlay	Tubotympanic disease	Tympanoplasty	cornea	graft ok	None
60	V	45	M	R	U/L	dry ear, Df	Inlay	Tubotympanic disease	Tympanoplasty	cornea	graft ok	None
61	R	23	F	L	U/L	dry ear, Df	Onlay	Tubotympanic disease	Tympanoplasty	cornea	graft ok	None
62	R	23	F	L	B/L	dry ear, Df	Inlay	Tubotympanic disease	Tympanoplasty	cornea	graft ok	None
63	U	33	F	L	U/L	dry ear, otalgia	Inlay	Tubotympanic disease	Tympanoplasty	cornea	graft ok	None

## Discussion

This pilot study has been undertaken as cornea and TM share lots of similarities, which can be considered as one of the graft materials for closure of TM perforation. Before diving into the literature data, below mentioned are the reasons why cornea is an apt graft material instead of TF for T-plasty.

TF remains the most frequently used graft material for closure of TM perforation, as it is easy to harvest, has a low basal metabolic rate similar to TM, is available in abundance for revision cases, and is at the same operating site. For reconstruction of a well-aerated tympanic cavity, TF fascia is the only suitable autogenous material with a success rate of 93-97% in primary T-plasty. Various materials have been used for closure of TM perforation for ages, including lobular fat, loose areolar fascia, fascia lata, dura mater, perichondrium, peritoneum, aortic valve, amniotic and mucous membrane, skin, pedicle skin, cornea, sclera, gelfilms, gelfoams, alloderm, cigarette paper, egg membrane, vein graft, periosteum & perichondrium from concha & tragus, and concha & tragal cartilage (2). 

The cornea has the same vertical and horizontal diameter of 11.5-11.6 mm with a thickness of 0.5-0.6 mm in the center and 1-1.2 mm at the periphery. The radius of curvature is 5 mm. Cornea that are rejected and not utilized for cornea transplantation are used for M-plasty. Corneal grafts can be either lamellar or penetrating. The lamellar graft includes epithelium, Bowman’s membrane, and stroma. It is a split-thickness corneal graft. The penetrating graft is a full-thickness graft that includes the entire cornea lined with epithelium and endothelium. Both homografts and heterografts of cornea were earlier used to improve vision or in therapeutic grafting of the eye (3).

Cornea substitutes not just the epithelial but also the fibrous layer of the drum, which later gets covered by an outgrowth of epithelium from surrounding tissues. Cornea is stable as a free graft and survives as a free graft until vascularization is established by ingrowth of vessels from the periphery. The smooth inside of the cornea is placed facing the promontory, which lessens the risk of adhesions. Finally, the cornea is resistant to necrosis, as it is nourished by diffusion from blood vessels and surrounding tissues in case an infection of the ME occurs (4).

The human cornea has 6 layers, making it a composite material. The thick middle layer has regularly arranged collagen fibers with interconnected keratocytes for general repair and maintenance. 90% of corneal thickness is composed of stroma, which has collagen and fibroblasts. The surface epithelium is composed of a stratified squamous, non-keratinizing, non-secretory layer. The Bowman’s layer is the acellular anterior condensation of stroma consisting of condensed collagen fibrils. Descemet's membrane is the posterior elastic lamina resistant to chemical agents, trauma, and pathological processes. The endothelial cells possess a water pump that keeps the cornea in a constant state of dehydration (5).

*Advantages of Cornea in T-plasty (* 6 *,* 7 *):*

- Diameter of cornea is 11.5 mm (TM is 8x10 mm).
- Thickness of cornea is 0.5-0.6 mm (TM is 0.1 mm) (aids in handling and positioning).
- The cornea borders with the sclera at the corneal limbus (TM inserted into the annulus tympanicus).
- The cornea is avascular except at the periphery (receives nutrition from capillary loops).
- Immunologically privileged tissue and can be used as a homologous graft.
- Lives by osmosis, is ectodermal in origin, and does not have a true endothelium.
- Transparency makes accurate positioning easy.
- Opacification is not concerned with its usage in ear surgery.
- Does not interfere with transmission of sound.

*Disadvantages of Cornea in T-plasty (8):*

- Human corneas are not readily available.
- Size of the cornea limits its use.

This review of literature mentioned below gives a comparison of various graft materials used in different studies so far in comparison to this study on a variety of parameters. 

As per a study conducted by Stanley (9), shield-sliced TC was used to close TM perforation in 223 pts, giving 98% successful graft uptake with preoperative ABG of 20 dB and postoperative ABG closure of 7 dB. Unlike this study, 88% successful graft uptake with corneal homograft preop ABG of 28 dB, postop ABG closure of 11 dB, and hearing gain of 16 dB among 63 pts.

60 pts over 2 years with TF and TP were the most preferred graft materials for closure of TM perforation. The study was based on parameters of graft uptake, audiological outcome, donor site complications, and late complications. The study showed that TP was found to have a better outcome than TF in terms of both graft uptake and hearing improvement, as per the study by Lebo (10). Unlike this study, where corneal homologous graft was considered effective with a success rate of 88% in uptake and 89% improvement in hearing.

220 pts of U/L CSOM with dry central perforation over 1 year were considered for surgery using autogenous graft materials (TF, TP, areolar tissue, and fat from earlobe) using the underlay technique between 13 and 48 years of age with a postop F/U for 6 months. Postoperative failure was due to upper respiratory infections and neglected postoperative advice and care, as per the study by Wright (11). Unlike this study, where T-plasty in cases with dry cases of CSOM (TTD) with transcanal technique and inlay approach was employed in 63 pts over 2 years between 18 and 60 years of age, showing 88% success on homologous corneal graft uptake with postop F/U having intervals of within 6 months, 7 months to 1 year, and more than a year, even with 7 pts undergoing revision surgery with other graft materials with failure to take up corneal graft.

M-plasty using the transcanal technique with the inlay approach in the ipsilateral ear was done with autologous fat from the earlobe, showing > 90% successful graft uptake over a study spread over 6 months. Unlike this study, where T-plasty with a similar transcanal technique and inlay approach was employed in 63 pts spread over a period of 2 years with 88% success on homologous corneal graft uptake as per the study by House (12).

According to a study by Forman (13), 93% of patients showed successful graft uptake using 2 autologous graft materials (TF and CP) for closure of central perforation in the inactive mucosal type of chronic otitis media, and 84% had successful hearing improvement within 20 dB in a study with 84 patients at the end of 12 weeks during postop F/U. Unlike this study, 63 pts underwent T-plasty with corneal homograft, having a dry ear, with 88% showing successful graft uptake with postopABG closure with a mean of 16 dB during postop F/U having intervals of within 6 months, 7 months to 1 year, and more than a year even.

56 pts underwent myringoplasty with sliced island TC and TP composite graft with successful graft uptake of 97.29% and postop ABG closure of 10 dB. Unlike this study, 63 pts underwent T-plasty with corneal homograft, with 88% showing successful graft uptake with postop ABG closure of 16 dB, as per studies by King (14) and Derlackey (15).

223 pts (98.2%) showed successful uptake of TF with sliced TC graft, with 4 pts showing graft failure. Among them, 28 pts with large perforations underwent slight technical modification in graft repair as per the study conducted by Smelzer (16). The study could not gather information on hearing improvement by postoperative ABG closure. Unlike this study, 88% showed successful corneal graft uptake even with a small sample size of 63 pts. 7 pts among them underwent graft repair with other materials after failure with corneal homograft. 89% showed hearing restoration with successful postop ABG closure.

As per the study by Filatov (17), a vein graft was used in 27 pts, with 23 pts (85%) showing successful graft uptake and 19 pts (33%) showing improved hearing restoration, unlike this study with 63 pts showing 88% successful corneal graft uptake with 89% hearing restoration.

TF vs. CG comparison where TF is the most common graft material used in ME surgery, while CG is one of the best innovative alternatives in place of TF due to its commonality in features and facts on average in all the above-mentioned reviews of literature. Successful graft uptake with TF is seen in about 93% of patients (with +/- 5%) with various factors taken into account along with all prerequisites followed, and hearing restoration in patients following surgery is seen in 82% (with +/- 3%). In comparison to this study, 88% of patients had successful graft uptake and 89% had hearing restoration following surgery, taking into account the criteria. As it is a pilot study, the success rates were beyond expectations at this point. Further, a study on the same topic with a sample of 330 pts is in process, and the success rates for graft uptake and hearing restoration are both having good expected results. Larger data and longer follow-up are needed to prove the efficacy of corneal homograft in terms of both the effectiveness of graft uptake and efficient hearing audibility. As the sample size in this study is only 63, the acquired results, though satisfactory, leave behind a possible query or probable chance of desired impact on a larger scale of population. Hence, a study based on the same objectives with a sample size of 330 pts is in process, which is showing promising results so far, where fresh corneal homografts are considered and a longer duration of F/U is used, as this pilot study was just a starter for the main course in the field of graft application in ME surgery. 

## Conclusion

**The** cornea and TM are two surfaces of the same coin based on anatomical characteristics and functional benefits. This study is one of a kind, where there is a promising amalgamation of eye and ear. It is a perfect example of when innovativeness meets ideas, so that “what was used to see can now be used to hear.” With advancement of medical science, there is always scope for new applicability and practicality to be adapted for today and in the future. Thereby, the cornea can definitely cater to positive and desirable “space” in the “face of T-plasty.”
